# In Vitro Fermentation of Browsable Native Shrubs in New Zealand

**DOI:** 10.3390/plants11162085

**Published:** 2022-08-10

**Authors:** James Chege Wangui, James P. Millner, Paul R. Kenyon, Peter R. Tozer, Patrick C. H. Morel, Sarah J. Pain

**Affiliations:** School of Agriculture and Environment, Massey University, Private Bag 11 222, Palmerston North 4442, New Zealand

**Keywords:** native shrubs, in vitro fermentation, volatile fatty acids, greenhouse gases, hill country

## Abstract

Information on the nutritive value and in vitro fermentation characteristics of native shrubs in New Zealand is scant. This is despite their potential as alternatives to exotic trees and shrubs for supplementary fodder, and their mitigation of greenhouse gases and soil erosion on hill-country sheep and beef farms. The objectives of this study were to measure the in vitro fermentation gas production, predict the parameters of the in vitro fermentation kinetics, and estimate the in vitro fermentation of volatile fatty acids (VFA), microbial biomass (MBM), and greenhouse gases of four native shrubs (*Coprosma robusta*, *Griselinia littoralis*, *Hoheria populnea*, and *Pittosporum crassifolium*) and an exotic fodder tree species, *Salix schwerinii*. The total in vitro gas production was higher (*p* < 0.05) for the natives than for the *S. schwerinii*. A prediction using the single-pool model resulted in biologically incorrect negative in vitro total gas production from the immediately soluble fraction of the native shrubs. However, the dual pool model better predicted the in vitro total gas production and was in alignment with the measured in vitro fermentation end products. The in vitro VFA and greenhouse gas production from the fermentation of leaf and stem material was higher (*p* < 0.05), and the MBM lower (*p* < 0.05), for the native shrubs compared to the *S. schwerinii*. The lower in vitro total gas production, VFA, and greenhouse gases production and higher MBM of the *S. schwerinii* may be explained by the presence of condensed tannins (CT), although this was not measured and requires further study. In conclusion, the results from this study suggest that when consumed by ruminant livestock, browsable native shrubs can provide adequate energy and microbial protein, and that greenhouse-gas production from these species is within the ranges reported for typical New Zealand pastures.

## 1. Introduction

Sheep are efficient utilizers of pastures due to the symbiotic anaerobic microbiota, which mainly comprises the bacteria, fungi, and protozoa in their reticulorumen [1,2,3]. These microorganisms obtain nutrients by fermentatively breaking down the ingested feed, and providing organic acids, microbial proteins, and some B complex vitamins to the host in return [4]. These fermentation processes also produce gases, primarily carbon dioxide and methane, which are major greenhouse gases [4]. Organic acids, principally acetic, propionic, and butyric volatile fatty acids (VFA) supply 70 to 80% of the dietary energy [4] of ruminants, while microbial protein provides 70 to 100% of the amino acids they require [5]. However, diet influences the reticulorumen microbial profile, which determines the rate of substrate fermentation and VFA and gas composition [4,6,7]. Fibrous diets are slow to degrade and encourage acetogenic anaerobes and methane-gas production, while diets rich in simple carbohydrates are highly fermentable and promote mainly propiogenic microbes and carbon-dioxide-gas production [6,7].

Both in vivo and in vitro methods can be used to evaluate feed substrates’ digestibility and their nutritive value, but in vitro methods are preferred because they are convenient and inexpensive proxies for in vivo fermentation [3,8]. In vitro methods involve the incubation of feed substrate in buffered rumen fluid with measurement of gas production periodically, and VFA and residue at the end of the fermentation process [4,9]. Unlike in vivo methods, in which VFAs are absorbed across the rumen wall, VFAs accumulate in rumen fluids in vitro, and their concentration indicates the actual production from the substrate [4]. Similar to in vivo methods, the residue obtained after the fermentation process can be used to determine the substrate digestibility and the microbial protein utilizable by the host [7].

Gas produced in vitro correlates with the rate of substrate fermentation and can be fitted to mathematical functions to estimate feed-substrate fermentation kinetic parameters [3,10,11]. Non-linear mathematical functions, mainly exponential and sigmoidal, are preferred because of their ability to model microbial growth and the fact that their parameters can be biologically explained [11,12,13]. However, the suitability of the mathematical function for the estimation of the fermentation kinetic parameters depends on the nutritive composition of the feed substrate [3,13,14]. Single-compartment models, also referred to as single-pool functions, assume a constant rate of fermentation and are mainly suited to feed substrates with homogenous nutrients, while dual-pool functions consider two rates of fermentation and are suitable for feed substrates with heterogenous nutrients [11,12,13,14]. The application of both single- and dual-pool non-linear functions is common in feed evaluation because natural feeds consumed by sheep contain a mixture of substrates that vary in nutrient composition [11].

Pasture, predominantly perennial ryegrass with a small proportion of clover, is the cheapest and most widely utilized feed resource in hill-country sheep and beef farms in New Zealand [15,16,17]. In addition, supplementary feeds are used during seasons with low pasture supply and quality [17,18]. The supplementary feeds that can be used on hill-country sheep and beef farms include conserved forages, alternative grazed forages, and concentrates [18]. Further, the foliage harvested from poplar and willow trees, commonly used for soil conservation on hill-country sheep and beef farms, can be used as a source of supplementary forage during the summer season [19,20,21]. Native shrubs may also offer potential feed sources when browsed in situ [22,23,24,25], but this has not yet been widely explored [22,23].

In New Zealand, native shrubs offer cultural value, genetic diversity, adaptability, and soil conservation, and they are evergreen and can supply foliage throughout the year [23,26]. Some native shrubs species are strongly preferred by wild herbivores, suggesting their potential as livestock-feed resources [24,25,27]. However, their nutritive value, in vitro fermentation gas production, fermentation kinetics, and fermentation end products in domesticated ruminants have not been previously studied. Information on the nutritive value, in vitro fermentation gas production, fermentation kinetics, and fermentation end products of New Zealand native shrubs could be used for comparison with conventional feed resources, which would aid in decision making by policy makers, researchers, and hill-country farmers. The objective of this study was therefore to determine the in vitro fermentation gas production, fermentation kinetics, and fermentation end products of four New Zealand native shrubs with potential use as sheep fodder in comparison to an exotic shrub utilized on North Island hill-country sheep and beef farms.

## 2. Materials and Methods

### 2.1. Shrubs and Study-Site Description

Four native shrub species, *Coprosma robusta* (Karamū), *Griselinia littoralis* (Pāpāuma), *Hoheria populnea* (Houhere), and *Pittosporum crassifolium* (Karo), and an exotic osier willow, *Salix schwerinii* (Kinuyanagi), were compared in this study. The five species were selected from among eight shrubs planted in August 2019 on a fenced trial site (lat −40.401447, long 175.617912) at the Massey University No. 4 Dairy farm, five kilometers south of Palmerston North. Other shrubs present but not included in the comparison were *Pseudopanax arboreus* and Hawke’s Bay ecotypes of *G. littoralis* and *P. arboreus*. They were not included due to insufficient herbage production.

The trial site was set up in a randomized complete block design, with four blocks. Each block had eight plots randomly allocated to shrub species or ecotype. Plots were planted with 15 shrubs in three rows and five columns and spaced at 1.5 m by 1.5 m. The trial site was on a southerward-aspect steep slope (>25°) dominated by Tokomaru silt loam soil at the top and Ohakea silt at the bottom [28]. Tokomaru silt loam and Ohakea silt loam are characterized by average natural fertility and fair-to-poor drainage, and are mainly used for pastoral farming [28]. Prior to planting, the site was used for dairy-cattle grazing. The climatic conditions for Palmerston North are defined in Table 1 [29].

### 2.2. Sample Collection and Processing

Foliage samples, consisting of leaved stems with a diameter of less than 5 mm, were collected in October 2020. Five shrubs in each plot were randomly selected and at least five foliage samples harvested from each shrub. A total of 20 foliage samples were collected, comprising five shrub species, in each of the four blocks. The foliage samples were indiscriminately harvested from the lower, middle, and top parts (not higher than 1.1 m) of the shrub to imitate the browsing behavior of sheep and to ensure the samples were representative of the entire shrub. Foliage samples for each shrub species from each plot were pooled, labeled, and chilled to approximately 4 °C while being processed. Any foreign materials, such as grass, dead leaves, and spider webs were removed from the collected samples (*n* = 20, i.e., five species by four pooled samples), before further separating the foliage samples into leaf (*n* = 20) and stem (*n* = 20) sub-samples. The leaf sub-sample included the leaf blade, stipules, buds, and petiole. The stem sub-sample included the woody and soft bark to which the leaves were attached. The sub-samples were frozen before submission to the Massey University Food and Nutrition Laboratory for freeze drying, grinding and proximate analysis of nutrients. A portion of the ground sub-samples (20 leaf and 20 stems; *n* = 40) was submitted to Alltech laboratories, Auckland, for in vitro fermentation analysis.

### 2.3. Proximate Analysis and In Vitro Digestibility

The foliage sub-sample dry-matter content (DM) was determined as the percentage of the weight remaining after moisture loss during freeze drying and was estimated using the AOAC 925.10, 930.15 calculation. Pyrolysis and combustion following AOAC 968.06 (Dumas) method was used to estimate total nitrogen, which was multiplied by 6.25 to estimate the crude protein (CP) content in the sub-samples. Ash content was determined by combusting the organic matter (OM) portion of the DM in a Furnace at 550 °C following the AOAC 942.05 (feed, meat) method. Fiber fractions were estimated following AOAC 2002.04 method for neutral detergent fiber (NDF) and AOAC 973.18 for acid-detergent fiber (ADF) and acid-detergent lignin (ADL) using the Fibertec system.

In vitro dry-matter digestibility (IVDMD) and organic matter digestibility (IVOMD) were estimated by treating samples with a neutral detergent solution and digesting with pepsin and fungal cellulase enzymes, as described by [31], and were expressed as percentage of the DM. Digestible organic content in the dry matter (IVDOMD) was calculated as a product of sub-sample OM (100-ash) and IVOMD and expressed as a percentage of the DM. The IVDOMD was used to derive the metabolizable energy (ME) in megajoules per kilogram of DM (MJ/kg DM) by multiplying by a factor of 0.16 [32]. Because IVDMD, IVOMD, and IVDOMD were used to estimate the ME, only the ME values are discussed.

### 2.4. Measuring In Vitro Fermentation Gas Production

In vitro fermentation was carried out using the Alltech IFM™ (Alltech, New Zealand) was to determine fermentation gas production, VFA, and microbial biomass (MBM). The procedure used is described by [33]. Each sub-sample (20 leaf and 20 stems; *n* = 40) was duplicated into 0.5-gram portions to be incubated in an in vitro medium made up of a mixture of rumen-fluid inoculum and buffer solution. Rumen fluid was collected from a fistulated lactating dairy cow in the morning approximately two hours after ad lib feeding on pasture and supplemented with grass and maize silage, 0.5 kg of molasses, and 1.5 kg pelleted dairy concentrate. The freshly collected rumen fluid was filtered through a double layer of cheese cloth to remove undigested material and was mixed with 5.6 L of McDougall bicarbonate buffer solution [34] and 250 mL of a reducing agent to make an in vitro medium with a 20:80 rumen fluid-to-buffer ratio. Each sub-sample portion was added into 100 mL of in vitro medium in 250-milliliter bottles and incubated at 39 °C for 48 h with gentle stirring and periodic pH checks [35]. Fermentation gas production was measured using an automated-pressure transducer and recorded continuously for 48 h [36]. The recorded gas production was used to estimate the in vitro fermentation kinetics for the shrub species.

### 2.5. Estimating the In Vitro Fermentation Kinetics Parameters

Nonlinear mathematical models are widely used to fit in vitro gas production to describe the fermentation kinetics of ruminant feeds [14]. The nonlinear models vary in structure, parameters, time zero behavior, lag period, points of inflection, and fermentation gas pools [3,14]. Descriptions of some nonlinear models commonly used to fit in vitro gas production can be found in [3,11,13,14]. However, the exponential and sigmoidal models are preferred because they are robust in relating gas production to microbial mass and substrate levels [11] and can be structured to accommodate more than one fermentation gas pool [14]. Single-pool models are better predictors of fermentation kinetics of simple substrates and are suggested for feeds with low fiber, while multipool models are better descriptors of heterogenous substrate and consider each substrate fraction independently, and they are recommended for feeds with high fiber [14]. Combined use of single and multipool models has been suggested when determining the best model to describe fermentation kinetics, where substrate fractions in the test feeds are unknown [14]. Exponential single-pool [37] (Equation (1)) and logistic dual-pool [11] (Equation (2)) models are extensively used and validated in fitting in vitro fermentation gas production [13,38] and were selected to describe the in vitro fermentation kinetics of the study shrub species.

Measured gas production for each sub-sample was combined for each shrub species and fitted using SAS non-linear procedure (Proc NLIN) to estimate model parameters that describe the rates and volumes of gas production from the sub-samples at given time $\left(t\right)$ in hours. In the single-pool model, total gas production ($V_{ors}$) in milliliters per gram of dry matter (mL/g DM) was the sum of gas production from the highly (*a*, mL/g DM) and slowly (*b*, mL/g DM) fermentable nutrients, produced at similar fermentation rates (c), expressed in percentage gas production per hour (%/h). Total gas production in the dual-pool model $\left(V_{\mathrm{Sch}},\;\mathrm{mL}/g\;\mathrm{DM}\right)$ was the sum of gas production from the fast pool ($V_{1}$, mL/g DM) portion and the slow pool ($V_{2}$, mL/g DM) produced from the highly fermentable and slowly fermentable substrate fractions, respectively. The $V_{1}$ and $V_{2}$ were assumed to have similar lag (L) times, but the rates of gas production ($C_{1}$, %/h) for $V_{1}$ and ($C_{2}$, %/h) for $V_{2}$ were different.

(1) $$V_{ors}=a+b\left(1-e^{-ct}\right)$$

(2) $$V_{Sch}=\left[\left(V_{1}/\left(1+e^{\left(2+4\times C_{1}\left(L-t\right)\right)}\right)\right)+\left(V_{2}/\left(1+e^{\left(2+4\times C_{2}\left(L-t\right)\right)}\right)\right)\right]$$

Shrub total gas production after 24 h for the single pool (*V*_24_, mL/g DM) and dual pool ($V_{1_{24}}$ and $V_{2_{24}}$, mL/g DM) was determined at *t* = 24 h for both models. The gas production half-life was assumed to be time (*t*, h) at which half of the total gas for each pool was produced [13]. Gas production half-life was estimated using the *t* function of the [37] (*T*_0.5_, h) (Equation (3)) and [11] ($V_{T_{0.5}}$, h) (Equation (4)) models.

(3) T0.5=−ln−0.5Vorsb+ab+1/c

(4) $$V_{T_{0.5}}=-\left(\ln\left(-0.5\right)/4c\right)+L+\left(1/2c\right),$$

where $V_{T_{0.5}}$ was $V_{1_{T}{}_{0.5}}$ or $V_{2_{T}{}_{0.5}}$.

Model performance was determined by regressing predicted (*x*-axis) against observed (*y*-axis) [13,39,40,41] gas production over 48 h. Regression residuals were used to determine the model’s goodness of fit and accuracy using root mean square error (RMSE) and mean absolute percentage error (MAPE) metrics [42,43]. Since RMSE units were similar to the regressed variables, low RMSE indicated high fitness of the models [43]. A MAPE lower than 5% showed excellent, 10 to 25% good, and greater than 25% very low and unacceptable model-prediction accuracy [42]. Adjusted coefficient of determination (adjusted R^2^) was used to indicate the proportion of variability explained by the models, with values close to one suggesting a stronger relationship between the predicted and observed gas-production values [43].

### 2.6. In Vitro Fermentation End Products

After 48 h of in vitro fermentation, the medium (Section 2.4) pH was measured before centrifuging. The supernatant was used to determine the VFA concentration, and the residues were used to estimate the sub-sample’s digestibility and the MBM. Volatile fatty acids (acetate, propionate, butyrate and valerate) and their isomers (isobutyrate and isovalerate) were recovered using the method suggested by [44], and their concentration was determined using the Agilent GC 7890 (Flame Ionization Detector, FID) gas chromatography system. Total VFAs were expressed in millimolar (mM) concentration produced per gram of dry matter (g DM) incubated while individual VFA and their isomers were expressed as the percentage of the total VFA. The concentration of individual VFA was used to balance theoretical fermentation equations to predict the volume (mL) and mass (g) of carbon dioxide (CO_2_) and methane (CH_4_) gases produced per g DM during the sub-samples fermentation [45]. Predicted fermentation gas proportions were pooled using their global-warming potential [46] to determine the CO_2_ equivalent (Eq CO_2_) emission potential of the shrub samples per g DM. The residue (undegraded sub-samples containing MBM) weight was used to determine the apparent digested DM (aDMD) [47], while the weight after solubilization of the MBM was used to estimate the true digested DM (DMD) [48] as a percentage of the DM (% DM). The MBM (mg/g DM) yield was estimated as the difference between aDMD and DMD weights [48]. In addition, the results of total VFA, MBM, and greenhouse gases were divided by the corresponding digested dry matter to estimate the in vitro fermentation end products in terms of DDM (Supplementary Material Tables S1 and S2). However, only the results in g DM are discussed because estimation of forage production, nutritional composition, and intake in ruminants are mainly in terms of DM.

### 2.7. Statistical Analysis

SAS software version 9.4 (SAS Institute, Cary, NC, USA) was used to carry out the statistical analysis. Analysis of variance in the general linear-model procedure (proc GLM) was used to compare the differences between the shrub samples’ means for the leaves and stems for the proximate nutrients and in vitro fermentation end products. The means were considered different if *p*> 0.05 and were separated using the Tukey method.

## 3. Results

### 3.1. Shrubs’ Nutritional Composition

The proximate nutritional composition of both the leaves and the stems differed (*p* < 0.05) between the shrub species, except for the NDF content in the leaves (*p* > 0.05) (Table 2). The leaf DM was similar (*p* > 0.05) in both the *P. crassifolium* and the *S. schwerinii*, where it was higher (*p* < 0.05) than in all the other species. The ash content was higher (*p* < 0.05) in the *H. populnea* and lower (*p* < 0.05) in the *S. schwerinii* leaves than in all the other species. The CP was similar (*p* > 0.05) in the *H. populnea* and *S. schwerinii* leaves, and higher (*p* < 0.05) than all the other species, which did not differ (*p* > 0.05). The *Salix schwerinii* leaves had higher (*p* < 0.05) ADF than the *H. populnea* and *P. crassifolium*, which were similar (*p* > 0.05), while the *C. robusta* and *G. littoralis* did not differ (*p* > 0.05) from the other species. The lignin was higher (*p* < 0.05) in the *G. littoralis* and lower (*p* < 0.05) in the *S. schwerinii* and *H. populnea*, while the *C. robusta* and *P. crassifolium* were intermediate and did not differ (*p* > 0.05) from the other species.

The *Salix schwerinii* and *P. crassifolium* had similar (*p* > 0.05) stem DM, which was higher (*p* < 0.05) than in the other species. The *Hoheria populnea* had higher (*p* < 0.05) and the *S. schwerinii* had lower (*p* < 0.05) stem ash than all other species. The stem CP was similar (*p* > 0.05) in the *H. populnea* and *S. schwerinii*, where it was higher (*p* < 0.05) than in the other species, which were not different (*p* > 0.05). The stem ADF and NDF were higher (*p* < 0.05) in the *H. populnea* and lower (*p* < 0.05) in the *C. robusta*, compared to the other species. Unlike in the leaves, the *S. schwerinii* had higher (*p* < 0.05) stem lignin content than all the other species, except the *G. littoralis*, which was comparable (*p* > 0.05) to the other species.

The leaves’ IVDMD was similar (*p* > 0.05) and higher (*p* < 0.05) for the native shrubs than for the *S. schwerinii*. *Salix schwerinii* leaves had lower (*p* < 0.05) IVOMD than the native shrubs (Table 3). However, the IVDOMD and ME were similar (*p* > 0.05) for the *H. populnea* and *S. schwerinii* leaves and lower (*p* < 0.05) than the other shrubs, which did not differ (*p* > 0.05). 

There were no differences (*p* > 0.05) in stem IVDMD between the species. However, the *C. robusta* had higher and *P. crassifolium* had lower stem IVOMD than the other shrubs, which did not differ from any other shrub. The stem IVDOMD and ME were similar (*p* > 0.05) for *C. robusta* and *S. schwerinii,* and higher (*p* < 0.05) than for the other species, except for *G. littoralis,* which was not different (*p* > 0.05) from any of the other shrubs.

### 3.2. In Vitro Fermentation Gas Production and Kinetics

#### 3.2.1. In Vitro Gas Production

The total gas production from in vitro fermentation for the leaf and stem differed (*p* < 0.05) between the species (Table 4). For both leaf and stem material, the *S. schwerinii* had lower (*p* < 0.05) gas production compared to the native shrub species. The gas production from the leaves was similar (*p* > 0.05) to that of the stem for *H. populnea* and *S. schwerinii*, but for the other species, the gas production was higher (*p* < 0.05) for the leaves than for the stems.

#### 3.2.2. Shrubs In Vitro Fermentation Kinetics

**Figure 1:**
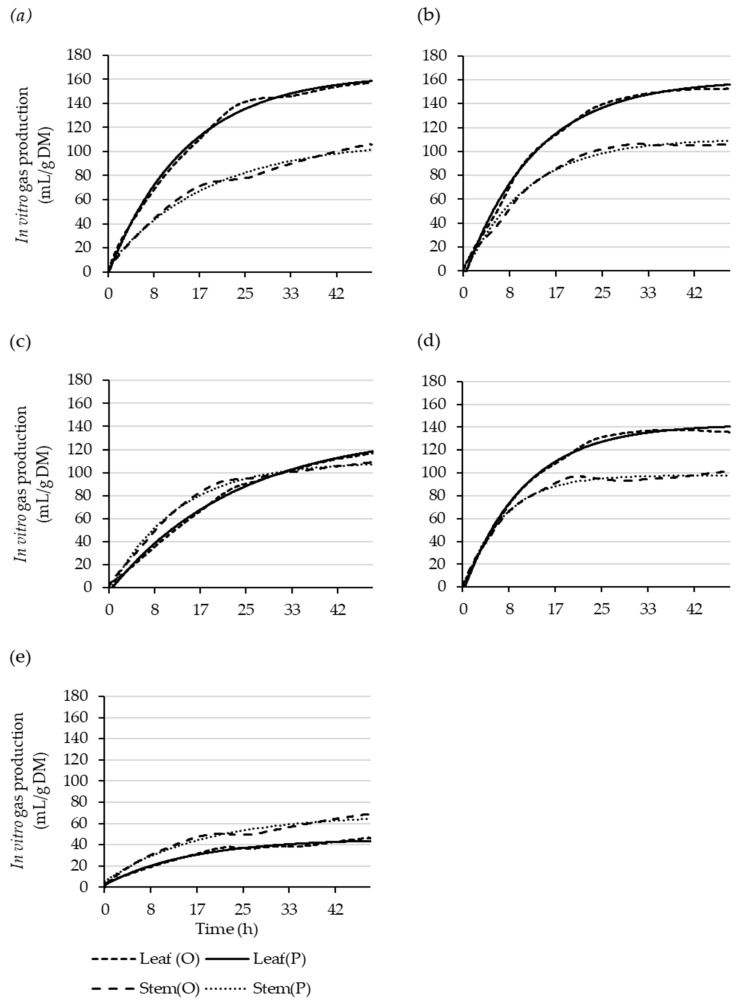
Observed (O) and single-pool-model-predicted (P) leaf and stem cumulative gas-production curves over 48 h for *Coprosma robusta* (**a**), *Griselinia littoralis* (**b**), *Hoheria populnea* (**c**), *Pittosporum crassifolium* (**d**), and *Salix schwerinii* (**e**).

The parameter estimates of the in vitro fermentation kinetics for the shrub leaves using the single-pool model are shown in Table 5, and the resulting gas-production curves are presented in Figure 1. The function parameters for the immediately soluble fraction (*a*, mL/g DM) were negative for the native shrubs. The gas production from the slowly degradable fraction (*b*, ml/g DM) and the total gas production (*V_ors_*, mL/g DM)) were more than three times higher in the native shrubs than in the *S. schwerinii*. However, the *H. populnea* had a slower rate of gas production (*c*, %/h) than all the other species, resulting in lower gas production after 24 h (*V*_24_, mL/g DM) and the longest gas production half-life (*T*_0.5_, h). The native shrub species had better model performance for the leaves than the *S. schwerinii*, which had very low accuracy (MAPE = 21.54) and a weak relationship (adjusted R^2^ = 0.414) between the observed and predicted gas production.

The parameter estimates for the stem in vitro fermentation kinetics (Table 6 and Figure 1) were negative, except in the *C. robusta* and *S. schwerinii*. The *b* and *V_ors_* parameters were higher in the natives than in the *S. schwerinii*. However, the *C. robusta* and *S. schwerinii* had lower *c*, resulting in lower *V*_24_ compared to the other shrub species. In contrast to the leaf model, the model for the *S. schwerinii* stems had the best fit (RMSE = 4.25) and explained a larger proportion of the variability (adjusted R^2^ = 0.935) between the predicted and observed gas production compared to the native shrub species, and had a high mean absolute percentage error (MAPE = 3.14).

**Figure 2:**
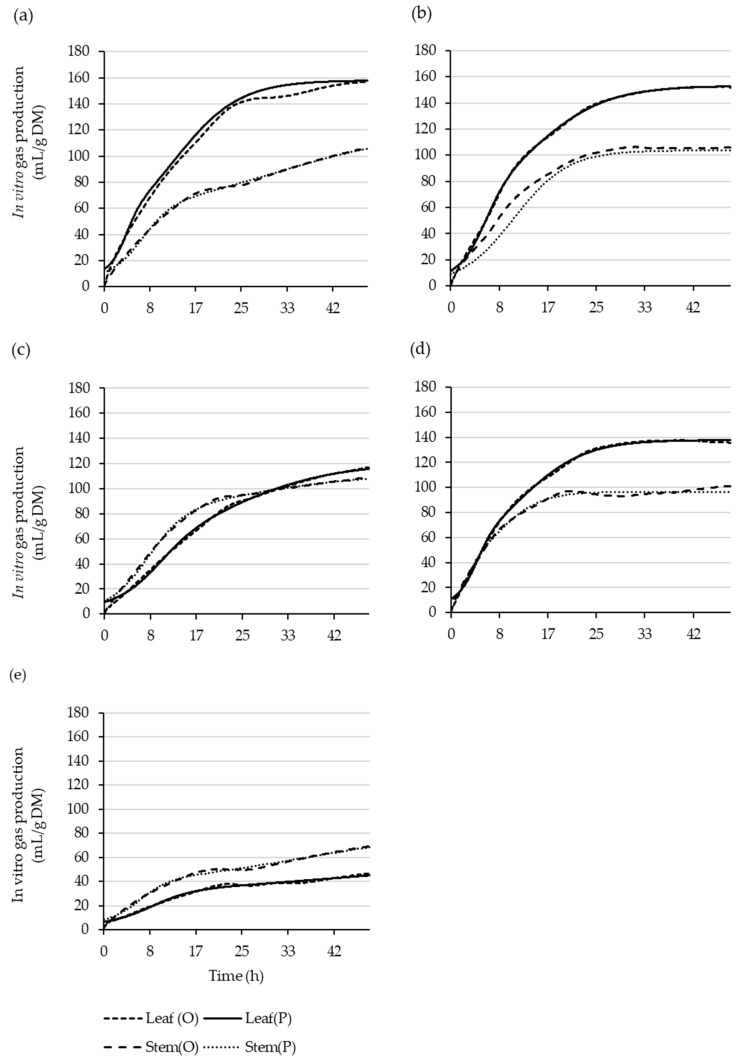
Observed (O) and the dual-pool-model-predicted (P) leaf and stem cumulative gas-production curves over 48 h for *Coprosma robusta* (**a**), *Griselinia littoralis* (**b**), *Hoheria populnea* (**c**), *Pittosporum crassifolium* (**d**), and *Salix schwerinii* (**e**).

The dual-pool-model in vitro fermentation kinetic parameter estimates for the leaves and stems are shown in Table 7 and Table 8, and the resulting predicted gas-production curves are presented in Figure 2. The predicted leaf gas production from the fast pool ($V_{1}$, mL/g DM) was more than three times higher in the native shrub species compared to the *S. schwerinii*. However, the *S. schwerinii* had the highest rate of gas production for the fast pool ($R_{1}$, %/h), resulting in a shorter gas-production half-life ($V_{1_{T_{0.5}}}$, h) compared to the native shrub species. The lag time (L, h) was shortest in the *S. schwerinii* and longest in the *H. populnea*. The gas production from the slow pool ($V_{2}$, mL/g DM) was higher in the natives compared to the *S. schwerinii*, except in the *C. robusta*. Unlike in the $V_{1}$, the *S. schwerinii* had the lowest rate of gas production for the slow pool ($R_{2}$, %/h), resulting in more than four times longer slow-pool gas-production half-life ($V_{2_{T_{0.5}}}$, h) compared to the native shrub species. The gas production after 24 h ($V_{1_{24}}\;$ and $V_{2_{24}}$, mL/g DM) was more than three times higher in the native shrub species compared to the *S. schwerinii*. Similarly, the performance of the dual -pool model was better for the native shrub species compared to the *S. schwerinii*, which had very low accuracy (MAPE = 22.42), and the relationship between the observed and predicted gas production was weaker (adjusted R^2^ = 0.417) (Table 7). Stem $V_{1}$ was lowest in the *H. populnea* compared to the other species. *Salix schwerinii* had the lowest $R_{1}$ but the shortest $V_{1_{T_{0.5}}}.$ Stem L was highest for the *C. robusta*, followed by the *S. schwerinii*, and lowest for the *P. crassifolium*. The *Hoheria populnea* had the highest $V_{2}$, followed by the *G. littoralis*, while the other species were similarly lower. The *Pittosporum crassifolium* had the highest $R_{2\;}$, resulting in the shortest $V_{2_{T_{0.5}}}$ compared to the *S. schwerinii*, which had more than four times longer $V_{2_{T_{0.5}}}$. The dual-pool model for the *S. schwerinii* stems had the best fit (RMSE = 3.71) and explained a larger proportion of the variability (adjusted R^2^ = 0.950) between the predicted and observed gas production compared to the native shrub species, and had a relatively good MAPE (−0.409) (Table 8).

### 3.3. In Vitro Fermentation End Products

The in vitro fermentation end products expressed on a DDM basis were more elevated than those on DM basis. However, the in vitro fermentation end products were found to have similar outcomes in the comparison of the native shrub species to the *S. schwerinii*, regardless of the analysis method. Therefore, the results are presented on a DM basis.

#### 3.3.1. Volatile Fatty Acids and Microbial Biomass

The pH of the in vitro medium after fermentation ranged from 6.57 to 6.71 and differed (*p* < 0.05) among species for the leaves but not (*p* > 0.05) the stems (Table 9). The leaf pH was higher (*p* < 0.05) in the *S. schwerinii* than in all the other species, except the *H. populnea*. For both the leaves and the stems, the *S. schwerinii* had nearly twice the (*p* < 0.05) MBM of the native shrub species, which did not differ (*p* > 0.05). 

The VFA varied (*p* < 0.05) among the species for the leaves and stems, except for the valerate in the leaves and the propionate and isovalerate in the stems. The *Coprosma robusta* and *H. populnea* were similar (*p* > 0.05) and had higher (*p* < 0.05) in vitro medium acetate than the *P. crassifolium* and *S. schwerinii*, which did not differ (*p* > 0.05). The *Salix schwerinii* leaves produced higher (*p* < 0.05) propionate and lower (*p* < 0.05) butyrate than the native shrub species However, the *H. populnea* and *P. crassifolium* leaves were similar (*p* > 0.05) and had higher (*p* < 0.05) butyrate and valerate isomers than the other species. Consequently, the ratio of acetate to propionate was similar (*p* > 0.05) for the *S. schwerinii* and *P. crassifolium*, where it was lower (*p* < 0.05) than in the other species. The total VFA produced from the in vitro fermentation of the leaf material was similar (*p* > 0.05) in the native shrub species and approximately four times higher (*p* < 0.05) than in the *S. schwerinii.* The *Salix schwerinii* stem in vitro medium had higher (*p* < 0.05) acetate and lower (*p* < 0.05) valerate than the other species, except the *C. robusta*, which was not different (*p* > 0.05) from any of the other species. The *Salix schwerinii* had lower (*p* < 0.05) butyrate in the stem than the other species. However, the isobutyrate was higher in the *H. populnea* stem than in the other species, except for the *P. crassifolium*. The total VFA production from the in vitro fermentation of the stem material was higher in the native shrub species (*p* < 0.05) than in the *S. schwerinii.*

#### 3.3.2. Fermentation Greenhouse Gases

The fermentative production of the greenhouse gases, carbon dioxide (CO_2_) and methane (CH_4_), and the carbon-dioxide equivalent (CO_2_ Eq) differed among the shrub species for both the leaf and stem samples (Table 10). The production of greenhouse gases and CO_2_ Eq was lower (*p* < 0.05) in the *S. schwerinii* than in the native shrub species for the leaf material. Similarly, the production of CO_2_ from the fermentation of the stem material was also lower (*p* < 0.05) for the *S. schwerinii* compared to the native shrub species. The production of CH_4_ from the fermentation of the stem material for the *S. schwerinii* was comparable (*p* > 0.05) to that of the *C. robusta* and *P. crassifolium*, and in relation to the production of CO_2_ Eq, the *S. schwerinii* stems were similar to the *P. crassifolium*.

## 4. Discussion

The objectives of the study were to (i) determine the in vitro fermentation gas production, (ii) predict the in vitro fermentation kinetics using the single- and dual-pool models, and (iii) to estimate the in vitro fermentation end products (volatile fatty acids and greenhouse gases) of four native shrubs with forage potential. A further objective was to compare the native shrubs to an exotic osier willow utilized on North Island hill-country sheep and beef farms. For the purposes of the discussion of the findings, the results sequence was rearranged from the order used in the Section 2 and Section 3.

### 4.1. Shrubs’ Volatile Fatty Acid and Microbial Biomass Production

Approximately 70% of the caloric requirements of ruminants are met by the volatile fatty acids (VFA) produced by reticulorumen microbes [49]. However, reticulorumen microbes’ metabolism and, thus, the quantity and proportions of the VFA produced, are affected by nutrients and non-nutritive factors in the diet [50,51]. The in vitro fermentation of the leaf and stem material from the native shrub species studied here resulted in more than three times the amount of total VFA (tVFA) compared to the *S. schwerinii*. The high tVFA yield in the native shrub species suggests that their nutrients were more digestible and could supply more ME to the animal than the *S. schwerinii.* The lower tVFA production during the in vitro fermentation of the *S. schwerinii* may have been due to the condensed tannins (CT), which are known to be present in Salix spp foliage [52,53,54]. However, the CT differences between the species in this study were not measured; therefore, this hypothesis cannot be tested and requires further attention.

Condensed tannins are complex polyphenolic compounds that bind to dietary proteins, polysaccharides, minerals, and microbial endogenous proteins and enzymes, thereby retarding microbial growth and proliferation and, hence, the production of VFA [55,56]. Although some pasture and fodder crops used in New Zealand contain small quantities of CT [57,58], which reduce in vitro VFA production [49], *S. schwerinii* foliage has been reported to contain higher levels (<50 g CT/kg DM) [54,59]. Further, the foliage CT concentration was found to be higher for *S. schwerinii* grown in hill country than on the fertile flat and rolling lands in New Zealand [54], an environment where farmers are likely to plant native species. Comparatively, the tVFA produced by the native species (24.5 to 28.8 mM) was within the range reported for perennial ryegrass–white-clover pastures with up to 25% chicory (24.5 to 27.2 mM) [60], higher than for tropical shrubs (8.9 to 20.8 mM) [33], and lower than for leguminous shrubs (73.2 to 97.2 mM) [61], pasture grasses (perennial rye-grass, tall fescue, Yorkshire fog, phalaris and paspalum), leaves (112.1 mM) and stems (105.4 mM) [62], and ryegrass–white-clover pastures with more than 25% chicory (29.8 to 33.4 mM) [60].

The in vitro fermentation of the native shrub species’ leaves produced higher tVFA than that of the stems, which contrasted with that of the *S. schwerinii*. This was likely due to the native shrub species having more digestible dry matter in their leaves than their stems, and is to be expected, because stems contain higher levels of structural carbohydrates than leaves. This observation can be supported by the observation of higher VFA production levels from the in vitro digested dry matter for the native shrub species than the *S. schwerinii*. The higher tVFA production from in vitro fermentation of the *S. schwerinii* stems compared to the leaves was likely due to the presence of CT in the leaves. The authors of [54] observed higher CT levels in the leaves of *S. schwerinii* compared to their stems.

The primary VFAs produced in the rumen are acetate, propionate, and butyrate, with valerate and branched-chain VFAs only found in small quantities [63]. The proportion of non-glucogenic (combined acetate and butyrate) VFA produced from the in vitro fermentation of leaves and stems from the studied shrub species ranged from 66 to 76% of the tVFA. This was within the typical ranges reported for forages (64 to 80%) in New Zealand [49,62,64,65], tropical shrubs (70.1 to 73.4%) [33], and leguminous (69.1 to 76.9%) and non-leguminous shrubs (73.4 to 79.6%) [61].

Proportionately, there was more acetate from the in vitro fermentation of the native species leaves than stems. By contrast, the in vitro fermentation of the *S. schwerinii* stems produced greater amounts of acetate compared to the leaves. Acetate is a lipogenic VFA that results from the fermentation of the structural carbohydrates (ADF and NDF) of forage and reduces with an increase in lignin content [55,63]. All the shrubs had more fiber (NDF and ADF) in their stems than in their leaves. Further, the lignin content was lower in the native species’ stems than in their leaves, but the opposite for the *S. schwerinii.* Thus, typically, more acetate would be expected after the fermentation of the fibrous stems compared to the leaves. However, the proportion of acetate after the in vitro fermentation of the native species’ stems was lower than from the leaves. In addition, more butyrate and propionate resulted from the in vitro fermentation of the native species’ stems than from their leaves. This suggests that the native species’ stems had more readily digestible carbohydrates than the leaves. This was likely because the shrubs were in a vegetative state and only new growth stems (i.e., less than 5 mm in diameter) were collected. Although they were not investigated, soluble and storage carbohydrates are typically high in new growth stems, and their fermentation results in elevated butyrate and propionate VFAs, respectively [50,51,66,67].

The in vitro fermentation microbial biomass (MBM) yield was higher in the *S. schwerinii* than in the native species for both the leaf and the stem material and showed an inverse relationship to the tVFA production. The production of VFA in vitro corresponds to the growth and turnover of MBM and the subsequent degradation of feed substrates [49]. However, the rate of growth and turnover of microbes depend on nutrient supply from the host diet and are affected by non-nutritive and inhibitory factors that hinder organic-matter digestibility [50,51]. In contrast to the native species, the high in vitro MBM and low tVFA yield observed in the *S. schwerinii* suggests that there was low growth and turnover of microbes. This may have been due to the inhibitory effects of the CT [54,62,68,69,70], which have been reported to be high in *S. schwerinii* [54,59]. Nutritionally, feedstuffs with low digestibility have been shown to have low reticulorumen microbe turnover and, hence, reduced microbial protein supply to animals [68,69,71].

### 4.2. In Vitro Gas Production

The in vitro gas production was higher in the native species than in the *S. schwerinii*. The lower in vitro gas production in the *S. schwerinii* may be attributed to the presence of CT. On average, the in vitro gas production from the native shrubs (112.5 to 131.2 mL/g DM) was within the range previously reported for leguminous shrubs (113.7 to 148.5 mL/g DM), and higher than for the non-leguminous shrubs (28.1 to 101.4 mL/g DM), but lower than for the *Moringa oleifera* (187.0 mL/g DM) [61] and ryegrass (193.0 mL/g DM) [66]. The shrubs’ in vitro gas production was consistent with the tVFA production and in inverse proportion to the MBM yield. This supports the results of previous studies, which showed a positive correlation between in vitro gas production and tVFA [72,73] and an inverse relationship with MBM yield [71].

The native species’ in vitro fermentation -gas production was higher in the leaves than in the stems, in contrast to the *S. schwerinii.* The high in vitro gas production in the native species leaves might have been associated with a higher production of acetate relative to butyrate and propionate VFAs [70], contrary to the stems. The stoichiometry of VFA proportions can be used to estimate the amount of gas production when feed substrates are fermented in vitro [71]. The fermentative formation of acetate has been reported to result in higher in vitro gas production [69,70], explaining the high in vitro gas produced by the native species’ leaves compared to their stems. Another factor that may have contributed to the higher in vitro gas production in the native species leaves is the higher CP content of the leaves compared to the stems. Dietary CP provides reticulorumen microbes with nitrogen, which is essential for growth and proliferation, enhancing carbohydrate degradation, resulting in increased gas production [70].

Among the native species, the *H. populnea* leaves had lower in vitro gas production despite having a higher CP. The lower in vitro gas production by the *H. populnea* leaves can likely be explained by the presence of higher ash content (11.6% DM) than in all the other shrubs. Ash content suggests the presence of minerals that are inorganic and unfermentable [69]. Similar ash content in *H. populnea* leaves has also been reported previously [74,75] and was within the range of that of forages [49], pasture grass (perennial rye-grass, tall fescue, Yorkshire fog, phalaris, and paspalum), and leaves (8.9 to 12.1% DM), but higher than for stems (5.5 to 8.9% DM) [62].

### 4.3. In Vitro Fermentation Kinetics

Mathematical non-linear models are essential tools that can be used to describe in vitro fermentation gas production using parameters that have biological interpretations [14]. The models vary in their complexity and differ in the equation structures and parameters (pools or compartments) that are applied in their predictions of in vitro fermentation gas production [13]. In this study, the single-pool exponential model of [37] and the dual-pool logistic model developed by [11] were applied to fit the in vitro fermentation gas production. Both the models ranked the predicted in vitro fermentation gas production for the shrubs’ leaves similarly to the measured in vitro fermentation total gas production. However, the models showed discrepancies in their ranking of the predicted in vitro fermentation gas production for *C. robusta*, *G. littoralis* and *H. populnea* stems. The stems of these three species had similar in vitro fermentation total gas production measurement and therefore the discrepancies between the models are likely due to their fixed inflection points, which affected the predicted rate and asymptotic gas production [13]. However, both the single- and dual-pool models had good predictive accuracy (MAPE) and explained a greater proportion of the variability (Adjusted R squared) between the measured and predicted in vitro fermentation gas production of the shrubs, except the *S. schwerinii* leaves. The lower accuracy and higher variability of the *S. schwerinii* leaves is likely to have been due to the inconsistently low gas production observed in the measured in vitro fermentation gas production.

The single-pool model produced negative prediction for the in vitro fermentation gas production from the immediately soluble fraction (*a,* mL/g DM) for the native species’ leaves. Similarly, negative *a* was predicted for the native species’ stems, except for the *C. robusta*. A negative *a* was a mathematical anomaly for the model, indicating negative gas production, which is biologically incorrect. This showed the mathematical limitations of the single-pool model in predicting the in vitro fermentation gas production for the native shrubs. Moreover, the single-pool model numerically overestimated the in vitro fermentation gas production of the native species, except the *H. populnea* stems. This can likely be attributed to the model assumption of a constant rate of fermentation [76,77,78,79], despite the fact that the native shrubs’ leaves had varying fermentable nutrients. Previous studies also demonstrated these limitations of using the single-pool model in predicting the in vitro fermentation gas production on feeds with mixed fermentable substrates [13,14,77].

The dual-pool model predicted higher in vitro fermentation gas production and longer gas-production half-life for the fast pool for the native species’ leaves, in contrast to the *S. schwerinii*. This observation suggests that the nutrients in the leaves of the native shrub species were readily fermentable, which is supported by the shrubs’ observed nutrient composition, tVFA, and total gas production in this study. However, the model predicted higher in vitro fermentation gas production from the slow pool for the *H. populnea* stems. This was expected, because the *H. populnea* stems contained higher structural carbohydrates (NDF and ADF) compared to the other shrubs. The higher in vitro fermentation gas production for fast pool and longer gas-production half-life for the slow pool for the *S. schwerinii* stems agreed with in vitro fermentation tVFA proportions and the measured total gas production in this study. The coherence of the dual-pool model in predicting the in vitro fermentation gas production for the studied shrubs supported earlier studies using different forages [3,9,13,14]. However, the model numerically underestimated the in vitro gas production for all the shrubs, except the *C. robusta* leaves and *H. populnea* stems, which it overestimated. Therefore, there is a need for a comparison of the dual-pool model with other multicompartment models to determine the model that can best describe the in vitro fermentation gas production for the studied shrubs.

### 4.4. Greenhouse-Gas Emissiosn from the Shrubs

The greenhouse-gas production was higher for the native shrub species than for the *S. schwerinii*. In addition, the native species’ leaves produced more greenhouse gases than the stems, while the opposite tendency was observed for the *S. schwerinii*. Proportionately, the in vitro production of CH_4_ gas was greater in the native species’ leaves than in their stems, while the opposite was true for the *S. schwerinii*. The CH_4_ production by the *S. schwerinii* leaves (8.6 mL/g DM) and stems (14.9 mL/g DM) were lower, while those of the native species’ leaves (37.8 to 46.0 mL/g DM) and stems (20.9 to 29 mL/g DM) were within the ranges reported for New Zealand pastures (17.6 to 58.5 mL/g DM) [80]. Averaging the leaves and stems, the native shrubs had more CH_4_ gas than the high-CT browse (18.3 to 29.2 mL/g DM), less than the *Moringa stenopetala*, and amounts within the range reported for low-CT shrubs (34.2 to 40.8 mL/g DM) [81]. On the other hand, the *S. schwerinii* had lower average leaf and stem CH_4_ gas than the low- and no-CT browse species and values within the range of high-CT browse species [81]. The low-enteric-fermentation greenhouse-gas production by the *S. schwerinii* may be attributed to the presence of CT. The effects of CT on the reduction in enteric-fermentation greenhouse-gas production have been studied previously for forages [57,82] and leguminous and non-leguminous shrubs [61,83,84].

Carbon dioxide (CO_2_) and methane (CH_4_) are the major enteric-fermentation greenhouse gases (GHGs) produced by ruminants [70]. Compared to CO_2_ gas, CH_4_ gas is more important because it is more potent, equating to 25 carbon-dioxide equivalents (CO_2_ Eq) in global-warming potential [70,73]. Further, enteric-fermentation CH_4_ gas accounts for approximately 84% of the gross CH_4_ emissions in New Zealand [85,86]. However, the amount and proportions of the enteric-fermentation gases produced are dependent on the nutritional composition of ruminant feeds, as this influences reticulorumen microbial populations and fermentation pathways [70,87]. A higher acetate proportion results in higher CH_4_ emissions [70,88,89], as was observed in the native species leaves. Acetogenesis causes the release of hydrogen ions, which are utilized by methanogenic reticulorumen microbes to reduce CO_2_, thereby releasing CH_4_ as metabolites [88,90]. The production of CH_4_ gas by methanogenic microbes results in a 2 to 12% loss of energy from ingested feeds [88,89,91,92]. On the other hand, butyrate and propionate formation were elevated from the in vitro fermentation of native species stems. Butyrate and propionate syntheses act as hydrogen sinks and compete for hydrogen ions with methanogenic rumen microbes, thereby reducing CH_4_ and promoting CO_2_-production metabolic pathways [87,89], explaining the depressed CH_4_ production in the in vitro fermentation of the native species’ stems.

## 5. Conclusions

The findings in the current study show that the fermentation of the leaf and stem material from the native shrub species resulted in higher in vitro total gas production than in the *S. schwerinii*. The single- and dual-pool models used to predict the in vitro fermentation total gas production for the shrubs had a satisfactory fit. However, the single-pool model was biologically incorrect in predicting negative in vitro total gas production from the immediately soluble fraction of the native shrubs. On the other hand, the dual-pool model predicted the in vitro fermentation total gas production better and was coherent with the measured in vitro fermentation end products. The native shrubs produced greater amounts of volatile fatty acids in the in vitro fermentation of the leaves and stems than the *S. schwerinii.* Conversely, the *S. schwerinii* yielded more microbial biomass from the in vitro fermentation of the leaves and stems than the native species. The in vitro fermentation characteristics of the native species’ leaves and stems suggest that they were more digestible and could provide more energy and microbial proteins to animals compared to the *S. schwerinii* if consumed. Comparing among the native shrubs, *H. populnea* leaves would be superior when consumed by providing higher levels of crude protein and yielding lower in vitro fermentation total gas production and emitting lower volumes of greenhouse gases. This study suggests that when consumed by ruminant livestock, native shrubs can provide adequate energy and microbial protein, and that the greenhouse-gas production from these species is generally within the ranges reported for typical New Zealand pastures. Further studies are required to determine animal preference and intake and to quantify GHG production in vivo.

## Figures and Tables

**Table 1:** Summarized long-term climatic conditions for Palmerston North district (Source [29,30]).

Climate Parameters		Season			
		Summer	Autumn	Winter	Spring
Temperature	Mean (°C)	17.4	13.8	9.0	12.4
Rainfall	Total (mm)	222.0	189.0	246.0	43.7
	Percentage of total rainfall (%)	25.0	21.0	27.0	26.0
Wind	Mean speed (km/h)	15.8	14.0	13.9	16.8
Solar radiation	Mean daily (MJ/m2/d)	21.1	11.0	6.7	15.9

**Table 2:** Native (*Coprosma robusta*, *Griselinia littoralis*, *Hoheria populnea*, and *Pittosporum crassifolium*) and exotic (*Salix schwerinii*) shrub species leaf (*n* = 20) and stem (*n* = 20) dry matter (DM, %) as a percentage of the fresh weight, and ash (Ash, %), crude protein (CP, %), neutral detergent fibre (NDF, %), acid detergent fibre (ADF, %) and lignin (Lignin, %) as percentages of the DM and metabolizable energy (ME, MJ/kg DM).

Shrub Species	DM	Ash	CP	NDF	ADF	Lignin
Leaf						
*Coprosma robusta*	39.3 ^b^	7.0 ^bc^	7.9 ^b^	37.4	22.4 ^ab^	9.6 ^ab^
*Griselinia littoralis*	31.5 ^c^	7.3 ^b^	6.2 ^b^	32.1	22.3 ^ab^	12.0 ^a^
*Hoheria populnea*	31.1 ^c^	11.6 ^a^	14.0 ^a^	37.6	20.2 ^b^	7.5 ^b^
*Pittosporum crassifolium*	43.0 ^a^	6.5 ^c^	6.2 ^b^	36.4	20.7 ^b^	9.3 ^ab^
*Salix schwerinii*	43.1 ^a^	4.9 ^d^	15.1 ^a^	36.3	24.6 ^a^	8.8 ^b^
Pooled SE	0.57	0.17	0.41	1.31	0.63	0.62
Stem						
*Coprosma robusta*	35.8 ^b^	6.4 ^bc^	4.7 ^b^	46.6 ^c^	36.7 ^b^	9.1 ^b^
*Griselinia littoralis*	32.1 ^b^	5.6 ^c^	4.2 ^b^	48.6 ^bc^	38.6 ^ab^	10.2 ^ab^
*Hoheria populnea*	36.0 ^b^	9.0 ^a^	8.3 ^a^	53.5 ^a^	41.5 ^a^	9.2 ^b^
*Pittosporum crassifolium*	47.4 ^a^	6.7 ^b^	4.2 ^b^	52.0 ^ab^	41.0 ^ab^	8.8 ^b^
*Salix schwerinii*	48.5 ^a^	3.7 ^d^	7.0 ^a^	48.9 ^bc^	38.4 ^ab^	11.6 ^a^
Pooled SE	1.05	0.23	0.37	0.95	1.07	0.38

Nutrient values with different superscripts in columns for leaf and stem are different at *p* < 0.05.

**Table 3:** Native (*Coprosma robusta, Griselinia littoralis, Hoheria populnea*, and *Pittosporum crassifolium*) and exotic (*Salix schwerinii*) shrub species leaf (*n* = 20) and stem (*n* = 20) in vitro dry-matter digestibility (IVDMD, % DM), in vitro digestible organic content in dry matter (IVDOMD, % DM), in vitro organic-matter digestibility (IVOMD, % DM) and metabolizable energy (ME, MJ/kg DM).

Shrub Species	IVDMD	IVOMD	IVDOMD	ME
Leaf				
*Coprosma robusta*	78.8 ^a^	82.3 ^a^	75.0 ^a^	12.0 ^a^
*Griselinia littoralis*	78.6 ^a^	82.0 ^a^	74.6 ^a^	11.9 ^a^
*Hoheria populnea*	77.4 ^a^	80.1 ^b^	71.2 ^b^	11.4 ^b^
*Pittosporum crassifolium*	78.4 ^a^	81.8 ^ab^	74.7 ^a^	12.0 ^a^
*Salix schwerinii*	73.7 ^b^	76.5 ^c^	70.4 ^b^	11.3 ^b^
SE	0.32	0.40	0.42	0.07
Stem				
*Coprosma robusta*	67.8	69.9 ^a^	63.7 ^a^	10.2 ^a^
*Griselinia littoralis*	66.3	68.4 ^ab^	62.5 ^ab^	10.0 ^ab^
*Hoheria populnea*	65.2	66.5 ^a,b^	60.0 ^b^	9.6 ^b^
*Pittosporum crassifolium*	64.2	65.9 ^b^	60.0 ^b^	9.6 ^b^
*Salix schwerinii*	66.9	69.4 ^ab^	63.9 ^a^	10.2 ^a^
SE	0.87	0.91	0.82	0.13

Values with different superscripts in leaf and stem columns are different at *p*< 0.05.

**Table 4:** Total gas production in milliliters per gram of dry matter (mL/g DM) from the in vitro fermentation of leaf (*n* = 20) and stem (*n* = 20) material from native (*Coprosma robusta*, *Griselinia littoralis*, *Hoheria populnea*, and *Pittosporum crassifolium*) and exotic (*Salix schwerinii*) shrub species.

Species	Leaf	Stem	SE
*Coprosma robusta*	157.0 ^a^	105.9 ^a,†^	6.92
*Griselinia littoralis*	151.7 ^ab^	105.9 ^a,†^	5.01
*Hoheria populnea*	116.6 ^b^	108.6 ^a^	7.01
*Pittosporum crassifolium*	135.3 ^ab^	100.8 ^a,†^	8.80
*Salix schwerinii*	46.1 ^c^	68.6 ^b^	6.58
SE	8.42	5.12	

^abc^ Mean total gas production for the shrubs in columns with different-letter superscripts differ significantly at *p* < 0.05. ^†^ Mean total gas production of leaves and stems in rows with symbol superscripts differ significantly at *p* < 0.05.

**Table 5:** Native (*Coprosma robusta, Griselinia littoralis, Hoheria populnea*, and *Pittosporum crassifolium*) and exotic (*Salix schwerinii*) shrub species leaf in vitro fermentation kinetic parameters derived using singlepool model, where: *a*, gas production from the immediately soluble fraction (mL/g DM); *b*, gas production from the slowly degradable fraction (mL/g DM); *c*, rate of gas production (%/h); *V_ors_*, total gas production (mL/g DM); *V*_24_, total gas production after 24 h (mL/g DM); and t_0.5,_ half-life of total gas production (h).

Species	Parameters	*a*	*b*	*c*	*V_ors_*	*V*_24_	*T*_0.5_	MAPE	RMSE	Adj R^2^
*Coprosma robusta*	Value	−1.0	165.3	0.070	164.3	133.1	10.2	−0.59	11.48	0.932
	SE	0.99	0.93	0.001						
	Lower 95% CI limit	−3	163.5	0.067						
	Upper 95% CI limit	0.9	167.1	0.072						
*Griselinia littoralis*	Value	−6.9	166.6	0.079	159.7	134.5	9.9	2.18	5.69	0.983
	SE	0.51	0.47	0.001						
	Lower 95% CI limit	−7.9	165.7	0.078						
	Upper 95% CI limit	−5.9	167.5	0.08						
*Hoheria populnea*	Value	−3.5	139.7	0.043	136.2	85.9	17.5	−1.65	11.86	0.892
	SE	0.91	1.44	0.001						
	Lower 95% CI limit	−5.3	136.8	0.040						
	Upper 95% CI limit	−1.7	142.5	0.045						
*Pittosporum crassifolium*	Value	−4.4	146.7	0.091	142.3	125.9	8.3	2.56	16.17	0.840
	SE	1.53	1.41	0.002						
	Lower 95% CI limit	−7.4	143.9	0.087						
	Upper 95% CI limit	−1.4	149.5	0.096						
*Salix schwerinii*	Value	2.4	43.2	0.064	45.6	36.3	9.2	−21.54	13.25	0.414
	SE	1.12	1.08	0.005						
	Lower 95% CI limit	0.2	41.1	0.054						
	Upper 95% CI limit	4.6	45.3	0.073						

RMSE, root mean square error. MAPE, mean absolute percentage error. Adj R^2^, adjusted R squared.

**Table 6:** Native (*Coprosma robusta*, *Griselinia littoralis*, *Hoheria populnea*, and *Pittosporum crassifolium*) and exotic (*Salix schwerinii*) shrub species stem in vitro fermentation kinetic parameters derived using single-pool model, where: *a*, gas production from the immediately soluble fraction (mL/g DM); *b*, gas production from the slowly degradable fraction (mL/g DM); *c*, rate of gas production (%/h); *V_ors_*, total gas production (mL/g DM); V_24_, total gas production after 24 h (mL/g DM); and t_0.5,_ half-life of total gas production (h).

Species	Parameters	*a*	*b*	*c*	*V* *_ors_*	*V*_24_	*T*_0.5_	MAPE	RMSE	Adj R^2^
*Coprosma robusta*	Value	4.9	104	0.055	108.9	81.2	11.0	−2.46	7.22	0.931
	SE	0.59	0.63	0.001						
	Lower 95% CI limit	3.7	102.7	0.053						
	Upper 95% CI limit	6.0	105.2	0.057						
*Griselinia littoralis*	Value	−4.5	115	0.09	110.5	97.3	8.6	−0.07	10.94	0.876
	SE	1.03	0.95	0.002						
	Lower 95% CI limit	−6.5	113.1	0.087						
	Upper 95% CI limit	−2.5	116.8	0.094						
*Hoheria populnea*	Value	−4.1	113.7	0.082	109.6	93.7	9.4	−0.18	11.01	0.875
	SE	1.0	0.92	0.002						
	Lower 95% CI limit	−6.1	111.9	0.079						
	Upper 95% CI limit	−2.1	115.5	0.085						
*Pittosporum crassifolium*	Value	−2.0	100.1	0.14	98.1	94.7	5.2	0.16	6.78	0.920
	SE	0.75	0.73	0.002						
	Lower 95% CI limit	−3.5	98.7	0.137						
	Upper 95% CI limit	−0.5	101.5	0.144						
*Salix schwerinii*	Value	5.6	62.9	0.057	68.5	52.4	9.3	3.14	4.25	0.935
	SE	0.35	0.36	0.001						
	Lower 95% CI limit	4.9	62.2	0.055						
	Upper 95% CI limit	6.3	63.6	0.059						

RMSE, root mean square error. MAPE, mean absolute percentage error. Adj R^2^, adjusted R squared.

**Table 7:** Native (*Coprosma robusta, Griselinia littoralis, Hoheria populnea*, and *Pittosporum crassifolium*) and exotic (*Salix schwerinii*) shrub species leaf in vitro fermentation kinetic parameters derived using dual-pool model, where: L, lag time (h); $V_{1}$, fast-pool total gas production (mL/g DM); $V_{2}$, slow pool (mL/g DM); $R_{1}$, fast-pool rate of gas production (%/h); $R_{2}$, slow rate (%/h); *V*_*Sch*_, total gas production (mL/g DM); $V_{1_{24}}$, total gas production for the fast pool after 24 h (mL/g DM); $V_{2_{24}}$ slow pool after 24 h (mL/g DM); $V_{1_{T_{0.5}}}$, fast-pool total-gas-production half-life (h); and $V_{2_{T_{0.5}}},\;$ slow-pool half-life (h).

Species	Parameters	$V_{1}$	$R_{1}$	L	$V_{2}$	$R_{2}$	*V*_*Sch*_	$V_{1_{24}}$	$V_{1_{T_{0.5}}}$	$V_{2_{24}}$	$V_{2_{T_{0.5}}}$	MAPE	RMSE	Adj R^2^
*Coprosma robusta*	Value	120.3	0.043	1.36	33.02	0.191	153.3	104.3	12.3	33.0	5.4	−8.29	11.32	0.934
	SE	1.86	0.001	0.18	1.94	0.019								
	Lower CI	116.7	0.042	1.01	29.2	0.154								
	Upper 95% CI limit	124.0	0.044	1.71	36.8	0.228								
*Griselinia littoralis*	Value	92.2	0.040	1.84	60.8	0.123	153.0	75.8	13.6	60.8	6.9	−3.66	5.31	0.985
	SE	1.43	0.001	0.07	1.54	0.004								
	Lower 95% CI limit	89.4	0.039	1.69	57.8	0.116								
	Upper 95% CI limit	95.0	0.041	1.98	63.8	0.130								
*Hoheria populnea*	Value	70.5	0.025	2.67	50.2	0.069	120.7	38.2	21.0	49.2	10.3	−8.76	11.83	0.892
	SE	3.82	0.002	0.27	5.05	0.005								
	Lower 95% CI limit	63.0	0.022	2.15	40.3	0.059								
	Upper 95% CI limit	78.0	0.029	3.2	60.1	0.080								
*Pittosporum crassifolium*	Value	91.2	0.046	1.41	46.7	0.167	137.9	81.9	11.7	46.7	5.7	−5.53	16.08	0.842
	SE	3.43	0.002	0.23	3.6	0.019								
	Lower 95% CI limit	84.4	0.043	0.96	39.7	0.130								
	Upper 95% CI limit	97.9	0.049	1.86	53.8	0.204								
*Salix schwerinii*	Value	27.6	0.066	1.01	34.6	0.011	62.2	27.1	8.7	9.2	46.3	−22.42	13.21	0.417
	SE	3.62	0.013	2.2	26.37	0.005								
	Lower 95% CI limit	20.5	0.042	−3.31	−17.1	0.000								
	Upper 95% CI limit	34.7	0.091	5.34	86.4	0.021								

RMSE, root mean square error. MAPE, mean absolute percentage error. Adj R^2^, adjusted R squared.

**Table 8:** Native (*Coprosma robusta, Griselinia littoralis, Hoheria populnea*, and *Pittosporum crassifolium*) and exotic (*Salix schwerinii*) shrub species leaf in vitro fermentation kinetic parameters derived using dual pool model, where, L, lag time (h); $V_{1}$, fast-pool total gas production (mL/g DM); $V_{2}$, slow pool (mL/g DM); $R_{1}$, fast-pool rate of gas production (%/h); $R_{2}$, slow rate (%/h); *V*_*Sch*_, total gas production (mL/g DM); $V_{1_{24}}$, total gas production for the fast pool after 24 h (mL/g DM); $V_{2_{24}}$, slow pool after 24 h (mL/g DM); $V_{1_{T_{0.5}}}$, fast-pool total-gas-production half-life (h); and $V_{2_{T_{0.5}}},\;$ slow pool half-life (h).

Species	Parameters	$V_{1}$	$R_{1}$	L	$V_{2}$	$R_{2}$	*V*_*Sch*_	$V_{1_{24}}$	$V_{1_{T_{0.5}}}$	$V_{2_{24}}$	$V_{2_{T_{0.5}}}$	MAPE	RMSE	Adj R^2^
*Coprosma robusta*	Value	71.9	0.017	1.71	50.8	0.095	122.8	27.7	28.9	50.8	7.7	−4.87	6.83	0.938
	SE	3.16	0.001	0.17	0.67	0.003								
	Lower 95% CI limit	65.8	0.016	1.38	49.5	0.089								
	Upper 95% CI limit	78.1	0.019	2.04	52.2	0.101								
*Griselinia littoralis*	Value	76.0	0.049	1.06	30.2	0.129	106.2	81.3	10.6	16.7	10.7	4.91	11.14	0.872
	SE	4.48	0.002	0.24	4.62	0.017								
	Lower 95% CI limit	67.2	0.045	0.59	21.1	0.095								
	Upper 95% CI limit	84.8	0.053	1.53	39.2	0.162								
*Hoheria populnea*	Value	33.9	0.021	1.03	78.1	0.073	112.0	16.5	23.0	77.4	8.4	−6.15	10.93	0.876
	SE	2.16	0.003	0.21	2.77	0.003								
	Lower 95% CI limit	29.7	0.016	0.62	72.7	0.066								
	Upper 95% CI limit	38.2	0.027	1.44	83.5	0.079								
*Pittosporum crassifolium*	Value	65.5	0.068	0.60	31.2	0.219	96.7	64.6	8.1	31.2	4.4	−2.11	6.70	0.922
	SE	2.06	0.002	0.13	2.11	0.019								
	Lower 95% CI limit	61.4	0.065	0.35	27.1	0.181								
	Upper 95% CI limit	69.5	0.071	0.84	35.3	0.257								
*Salix schwerinii*	Value	56.2	0.014	1.42	32.6	0.097	88.8	32.5	7.8	18.0	36.5	−4.09	3.71	0.950
	SE	4.10	0.001	0.22	0.44	0.003								
	Lower 95% CI limit	48.2	0.012	0.99	31.7	0.092								
	Upper 95% CI limit	64.2	0.015	1.86	33.4	0.103								

RMSE, root mean square error. MAPE, mean absolute percentage error. Adj R^2^, adjusted R squared.

**Table 9:** The pH, total volatile fatty acids (VFA) in millimoles (tVFA, mM), percentage of respective VFA (scetate, propionate, isobutyrate, butyrate, isovalerate, valerate, %), ratio of acetate to propionate (A:P), and microbial biomass in milligrams per gram of dry matter (MBM, mg/g DM) for leaves and stems for native (*Coprosma robusta, Griselinia litoralis, Hoheria populnea,* and *Pittosporum crassifolium*) and an exotic (*Salix schwerinii*) shrub species with potential use as fodder sources in New Zealand.

Shrub Species	pH	Acetate	Propionate	Isobutyrate	Butyrate	Isovalerate	Valerate	Total VFA	A:P	MBM
Leaves										
*Coprosma robusta*	6.57 ^b^	63.1 ^a^	22.2 ^c^	0.15 ^b^	13.8 ^a^	0.11 ^b^	0.69	27.5 ^a^	2.9 ^a^	101.3 ^b^
*Griselinia litoralis*	6.57 ^b^	61.2 ^ab^	23.8 ^c^	0.00 ^c^	15.1 ^a^	0.00 ^b^	0.63	27.6 ^a^	2.6 ^a^	80.2 ^b^
*Hoheria populnea*	6.62 ^ab^	62.7 ^a^	25.0 ^bc^	0.38 ^a^	10.4 ^b^	0.58 ^a^	0.89	24.5 ^a^	2.5 ^a^	106.4 ^b^
*Pittosporum crassifolium*	6.57 ^b^	57.6 ^b^	27.6 ^b^	0.33 ^a^	13.1 ^a^	0.52 ^a^	0.92	28.8 ^a^	2.1 ^b^	112.5 ^b^
*Salix schwerinii*	6.70 ^a^	58.1 ^b^	33.9 ^a^	0.00 ^c^	7.9 ^c^	0.11 ^b^	0.75	7.3 ^b^	1.7 ^b^	260.2 ^a^
Pooled SE	0.027	0.98	0.73	0.033	0.57	0.062	0.070	0.99	0.09	9.88
Stem										
*Coprosma robusta*	6.66	60.4 ^ab^	25.0	0.00 ^b^	14.2 ^a^	0.00	0.74 ^ab^	17.4 ^a^	2.5	82.5 ^b^
*Griselinia litoralis*	6.67	54.7 ^b^	27.7	0.04 ^b^	17.6 ^a^	0.09	0.82 ^a^	19.0 ^a^	2.0	62.6 ^b^
*Hoheria populnea*	6.66	55.8 ^b^	27.9	0.29 ^a^	15.1 ^a^	0.38	1.03 ^a^	19.9 ^a^	2.0	70.1 ^b^
*Pittosporum crassifolium*	6.67	53.3 ^b^	31.9	0.09 ^ab^	13.9 ^a^	0.32	0.84 ^a^	16.0 ^a^	1.8	90.0 ^b^
*Salix schwerinii*	6.71	68.6 ^a^	25.9	0.00 ^b^	7.7 ^b^	0.00	0.29 ^b^	9.6 ^b^	2.7	144.4 ^a^
Pooled SE	0.013	2.04	2.08	0.054	1.39	0.091	0.115	1.28	0.22	7.22

VFA, A:P and MBM with different superscripts in a column for the sample type are different at *p*< 0.05. Sample VFA value of 0.00 indicates the VFA was undetectable.

**Table 10:** Native shrubs (*Coprosma robusta, Griselinia litoralis, Hoheria populnea,* and *Pittosporum crassifolium*) and an exotic (*Salix schwerinii*) shrub species carbon dioxide (CO_2_) and methane (CH_4_) gas production in milliliters per gram of dry matter (mL/g DM) and greenhouse carbon-dioxide equivalent (CO_2_ Eq) in grams per gram of dry matter (g/g DM) from the leaves and stems.

Shrub Species	CO_2_	CH_4_	CO_2_ Eq
Leaves			
*Coprosma robusta*	76.5 ^ab^	46.0 ^a^	0.77 ^a^
*Griselinia litoralis*	76.3 ^ab^	43.9 ^a^	0.77 ^a^
*Hoheria populnea*	64.2 ^b^	37.8 ^a^	0.66 ^a^
*Pittosporum crassifolium*	77.7 ^a^	41.3 ^a^	0.74 ^a^
*Salix schwerinii*	17.2 ^c^	8.6 ^b^	0.15 ^b^
Pooled SE	3.01	2.07	0.039
Edible stem			
*Coprosma robusta*	47.6 ^a^	26.7 ^ab^	0.45 ^a^
*Griselinia litoralis*	53.7 ^a^	27.1 ^a^	0.47 ^a^
*Hoheria populnea*	54.7 ^a^	28.0 ^a^	0.49 ^a^
*Pittosporum crassifolium*	42.1 ^a^	20.9 ^ab^	0.36 ^ab^
*Salix schwerinii*	23.5 ^b^	14.9 ^b^	0.25 ^b^
Pooled SE	3.54	2.76	0.031

Fermentation gas (CO_2_ and CH_4_) and carbon-dioxide equivalent (CO_2_ Eq) with different superscripts in a column for the sample type are different at *p*< 0.05.

## Data Availability

Data used in the study is available on request to the authors.

## Supplementary Materials

The following supporting information can be downloaded at: https://www.mdpi.com/article/10.3390/plants11162085/s1, Table S1: pH, total volatile fatty acids (VFA) in millimoles (tVFA, mM) from digested dry matter, percentage of respective VFA (Acetate, Propionate, Isobutyrate, Butyrate, Isovalerate, Valerate, %), ratio of Acetate to Propionate (A:P) and microbial biomass in milligram per gram of digested dry matter (MBM, mg/g DDM) for leaf and stem for native (Coprosma robusta, Griselinia litoralis, Hoheria populnea and Pittosporum crassifolium) and an exotic (Salix schwerinii) shrub species with potential use as fodder sources in New Zealand, Table S2: Native shrubs (Coprosma robusta, Griselinia litoralis, Hoheria populnea and Pittosporum crassifolium) and an exotic (Salix schwerinii) shrub species carbon dioxide (CO_2_) and methane (CH4) gas production in milliliters per gram of digested dry matter (mL/g DDM) and green house carbon dioxide equivalent (CO_2_ Eq) in grams per gram of digested dry matter (g/g DDM) from the leaf and stem.
