# Supplementary material for: In Vitro Fermentation of Browsable Native Shrubs in New Zealand
Source: Plants (Basel). 2022 Aug 10;11(16):2085. doi: 10.3390/plants11162085 (PMC9416311; doi:10.3390/plants11162085)
Supplement: Supplementary file 1 [file plants-11-02085-s001.zip › plants-1822879-supplementary.pdf]

Supplementary Table S1. pH, total volatile fatty acids (VFA) in millimoles (tVFA, mM) from digested dry matter, percentage of respective VFA (Acetate, Propionate, Isobutyrate, Butyrate, Isovalerate, Valerate, %), ratio of Acetate to Propionate (A:P) and microbial biomass in milligram per gram of digested dry matter (MBM, mg/g DDM) for leaf and stem for native (Coprosma robusta, Griselinia litoralis, Hoheria populnea and Pittosporum crassifolium) and an exotic (Salix schwerinii) shrub species with potential use as fodder sources in New Zealand.

**Shrub species**	**pH**	**Acetate**	**Propionate**	**Isobutyrate**	**Butyrate**	**Isovalerate**	**Valerate**	**Total VFA**	**A:P**	**MBM**
					Leaf					
Coprosma robusta	6.57^{b}	63.1^{a}	22.2^{c}	0.15^{b}	13.8^{a}	0.11^{b}	0.69	30.9^{b}	2.9^{a}	114.7^{c}
Griselinia litoralis	6.57^{b}	61.2^{ab}	23.8^{c}	0.00^{c}	15.1^{a}	0.00^{b}	0.63	43.7^{a}	2.6^{a}	127.1^{bc}
Hoheria populnea	6.62^{ab}	62.7^{a}	25.0^{bc}	0.38^{a}	10.4^{b}	0.58^{a}	0.89	27.1^{b}	2.5^{a}	117.4^{bc}
Pittosporum crassifolium	6.57^{b}	57.6^{b}	27.6^{b}	0.33^{a}	13.1^{a}	0.52^{a}	0.92	43.2^{a}	2.1^{b}	169.2^{b}
Salix schwerinii	6.70^{a}	58.1^{b}	33.9^{a}	0.00^{c}	7.9^{c}	0.11^{b}	0.75	8.2^{c}	1.7^{b}	291.6^{a}
Pooled SE	0.027	0.98	0.73	0.033	0.57	0.062	0.070	1.29	0.09	12.18
					Stem					
Coprosma robusta	6.66	60.4^{ab}	25.0	0.00^{b}	14.2^{a}	0.00	0.74^{ab}	27.0^{ab}	2.5	128.0^{bc}
Griselinia litoralis	6.67	54.7^{b}	27.7	0.04^{b}	17.6^{a}	0.09	0.82^{a}	22.1^{b}	2.0	73.0^{c}
Hoheria populnea	6.66	55.8^{b}	27.9	0.29^{a}	15.1^{a}	0.38	1.03^{a}	33.6^{a}	2.0	118.7^{c}
Pittosporum crassifolium	6.67	53.3^{b}	31.9	0.09^{ab}	13.9^{a}	0.32	0.84^{a}	32.9^{a}	1.8	185.3^{b}
Salix schwerinii	6.71	68.6^{a}	25.9	0.00^{b}	7.7^{b}	0.00	0.29^{b}	19.7^{b}	2.7	295.8^{a}
Pooled SE	0.013	2.04	2.08	0.054	1.39	0.091	0.115	2.38	0.22	13.46

VFA, A:P and MBM with different superscripts in a column for the sample type are different at $p < 0.05$. Sample VFA value of 0.00 indicate the VFA was undetectable.

Supplementary Table S2. Native shrubs (*Coprosma robusta, Griselinia litoralis, Hoheria populnea* and *Pittosporum crassifolium*) and an exotic (*Salix schwerinii*) shrub species carbon dioxide (CO_2) and methane (CH_4) gas production in milliliters per gram of digested dry matter (mL/g DDM) and green house carbon dioxide equivalent (CO_2 Eq) in grams per gram of digested dry matter (g/g DDM) from the leaf and stem

Species	$mLCO_2$ /gDDM	$mLCH_4$ /gDDM	gCO_2 Eqv /gDDM
	Leaf		
Coprosma robusta	86.1^{a}	51.8^{a}	0.870^{a}
Griselinia littoralis	84.3^{ab}	48.5^{a}	0.852^{a}
Hoheria populnea	72.0^{b}	42.3^{a}	0.741^{a}
Pittosporum crassifolium	90.2^{a}	48.0^{a}	0.858^{a}
Salix schwerinii	35.3^{c}	17.7^{b}	0.316^{b}
SE	3.16	2.20	0.04
	Stem		
Coprosma robusta	75.0^{a}	41.9	0.719^{a}
Griselinia littoralis	80.3^{a}	40.4	0.703^{a}
Hoheria populnea	84.8^{a}	43.3	0.752^{a}
Pittosporum crassifolium	70.4^{a}	34.7	0.601^{ab}
Salix schwerinii	48.0^{b}	30.5	0.510^{b}
SE	4.62	3.80	0.04

Fermentation gas (CO_2 and CH_4) and carbon dioxide equivalent (CO_2 Eq) with different superscripts in a column for the sample type are different at $p < 0.05$.
